# Differential alternative splicing regulation among hepatocellular carcinoma with different risk factors

**DOI:** 10.1186/s12920-019-0635-z

**Published:** 2019-12-20

**Authors:** Young-Joo Jin, Seyoun Byun, Seonggyun Han, John Chamberlin, Dongwook Kim, Min Jung Kim, Younghee Lee

**Affiliations:** 1 Department of Biomedical Informatics, University of Utah School of Medicine, Salt Lake City, UT USA; 2 Division of Gastroenterology, Department of Internal Medicine, Inha University School of Medicine, Incheon, South Korea; 3 Pharmacy program, Massachusetts College of Pharmacy and Health Sciences, Worcester, MA USA; 4 Huntsman Cancer Institute, University of Utah School of Medicine, Salt Lake City, UT USA

**Keywords:** Hepatocellular carcinoma, Alternative splicing, hepatitis B virus, Hepatitis C virus, Alcohol, RNA-sequencing

## Abstract

**Background:**

Hepatitis B virus (HBV), hepatitis C virus (HCV), and alcohol consumption are predominant causes of hepatocellular carcinoma (HCC). However, the molecular mechanisms underlying how differently these causes are implicated in HCC development are not fully understood. Therefore, we investigated differential alternative splicing (AS) regulation among HCC patients with these risk factors.

**Methods:**

We conducted a genome-wide survey of AS events associated with HCCs among HBV (*n* = 95), HCV (*n* = 47), or alcohol (*n* = 76) using RNA-sequencing data obtained from The Cancer Genome Atlas.

**Results:**

In three group comparisons of HBV vs. HCV, HBV vs. alcohol, and HCV vs. alcohol for RNA seq (ΔPSI> 0.05, FDR < 0.05), 133, 93, and 29 differential AS events (143 genes) were identified, respectively. Of 143 AS genes, eight and one gene were alternatively spliced specific to HBV and HCV, respectively. Through functional analysis over the canonical pathways and gene ontologies, we identified significantly enriched pathways in 143 AS genes including immune system, mRNA splicing-major pathway, and nonsense-mediated decay, which may be important to carcinogenesis in HCC risk factors. Among eight genes with HBV-specific splicing events, *HLA-A, HLA-C*, and *IP6K2* exhibited more differential expression of AS events (ΔPSI> 0.1). Intron retention of *HLA-A* was observed more frequently in HBV-associated HCC than HCV- or alcohol-associated HCC, and intron retention of *HLA-C* showed vice versa. Exon 3 (based on ENST00000432678) of *IP6K2* was less skipped in HBV-associated in HCC compared to HCV- or alcohol-associated HCC.

**Conclusion:**

AS may play an important role in regulating transcription differences implicated in HBV-, HCV-, and alcohol-related HCC development.

## Background

Hepatocellular carcinoma (HCC) continues to impose a burden on health care system worldwide, and is the 6th most common cancer and the 2nd most common cause of cancer-related death in the world [1]. The predominant risk factors (RFs) of HCC development are chronic hepatitis B virus (HBV) or hepatitis C virus (HCV) infection, and chronic alcohol consumption [2–4]. The pathogenesis of HCC involves a multistep process that includes hepatocyte inflammation or proliferation and genetic or epigenetic changes [5], and these three RFs are also known to affect HCC development by different mechanisms [6–9]. However, differences in molecular mechanisms underlying how these causes are implicated in HCC development remain largely unexplored.

The exact oncogenic mechanisms of HBV-related HCC are not fully understood [10–12], but it has been increasingly reported direct and indirect mechanisms are implicated in HBV-induced hepatocarcinogenesis. As regards the direct mechanism, HBV integration into the hepatocyte genome [6, 7, 13] may be involved in hepatocarcinogenesis through the induction of hepatocyte transformation. On the other hand, HBV may indirectly initiate the carcinogenic process by causing necroinflammation, regenerative hyperplasia, and fibrosis in hepatocyte, and consequently liver cirrhosis (LC) [7, 14, 15]. The major mechanism of HCV-related HCC is believed to be due to the indirect effects of HCV, that is, hepatocyte inflammation and proliferation, the inductions of genomic mutations and instabilities, mitochondrial damage to induce reactive oxygen species, and avoidance of host’s immune response [9, 16, 17]. Alcohol-related HCC is considered to be the result of hepatocyte necroinflammation and regeneration, oxidative stress, and LC [8]. These different hepatocarcinogenesis related to the three RFs make it difficult to manage HCC patients uniformly. Recently, a genomic landscape of HCC development was provided by next-generation sequencing (NGS), that is, whole genome, exome, or RNA sequencing [18–20]. Furthermore, alternative splicing (AS) events in the process of mRNA expression are known to produce different protein from single genes, and this has also been reported to occur during HCC development [21, 22].

Various changes at genome level and a few pathways are known to be associated with HCC development with each risk factor [23]. Thus, it is important to elucidate three risk factor-wide pathogenesis of HCC development at the systems level. More specifically, it is crucial to understand how these HCC-related genes are differently expressed. To date, however, no clear-cut HBV-, HCV-, or alcohol-related genetic or epigenetic profiles have been reported in HCC. Recent study showed that HBV- and HCV-associated HCCs are associated with altered AS events [22], but the process of extracting study subjects from The Cancer Genome Atlas (TCGA) was not described. Considering that the TCGA database contains mixed typed hepatocholangiocarcinoma, fibrolamellar carcinoma, patients ≤18 years, and recurrent HCC, these factors needs to be sorted out. Moreover, alcohol consumption, that is one of the significant RFs for HCC, need to be properly considered in the study.

In the present study, therefore, we conducted a genome-wide analysis to identify differential AS events among HBV-, HCV-, and alcohol-associated primary HCCs using RNA-Seq data for tumor tissues obtained after proper sorting of samples from the TCGA database. In addition, we showed the HCC development related pathway that may be affected by AS events.

## Methods

### Study subjects

**Fig. 1:**
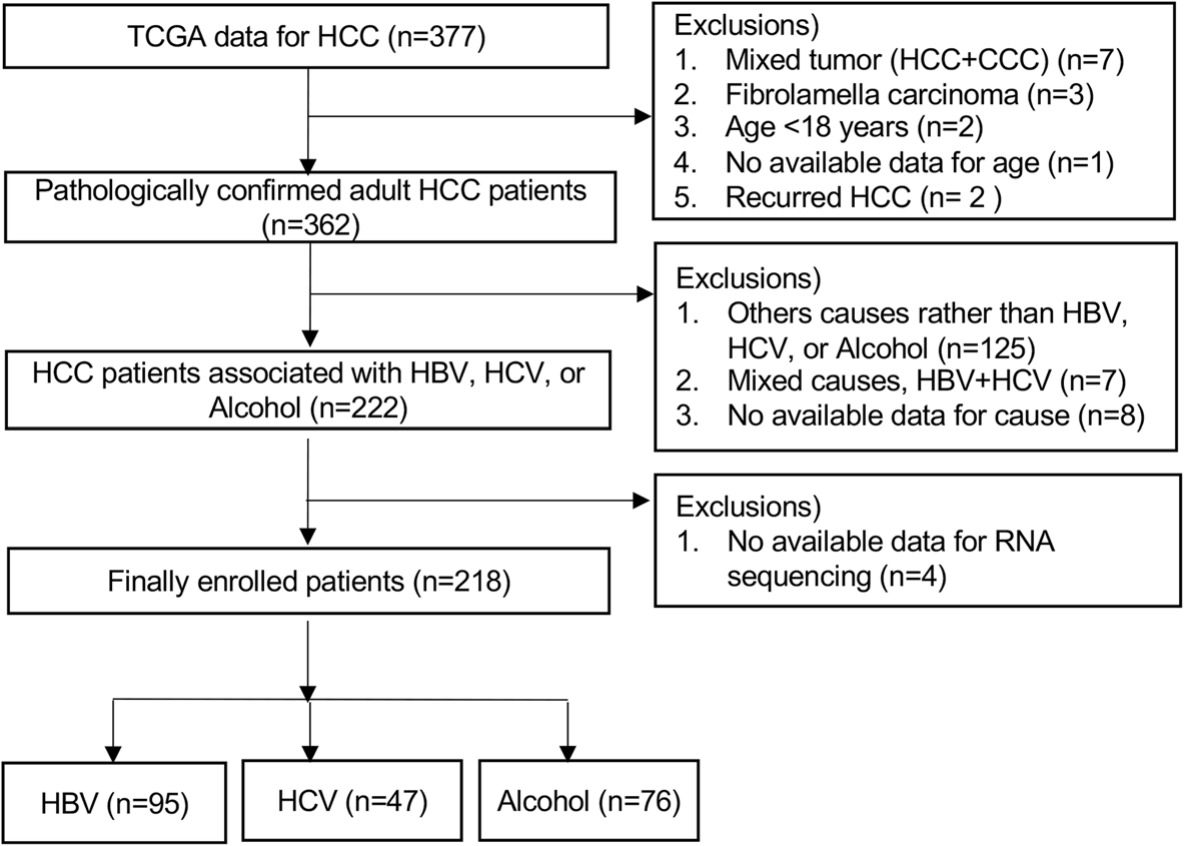
Flowsheet of the enrolled patients. Of the 377 patients, 218 patients were enrolled in the study

We retrospectively reviewed the clinical and genetic tumor data of 377 patients with liver cancer obtained from TCGA (Fig. 1). They underwent surgical resection (*n* = 376) or liver transplantation (*n* = 1) for liver cancer between 1995 and 2013.Of these 377 patients, 15 patients with mixed typed hepatocholangiocarcinoma (*n* = 7), fibrolamellar carcinoma (*n* = 3), aged ≤18 years (*n* = 2), no available data for age (*n* = 1), or recurrent HCC (*n* = 2) were excluded. Of the remaining 362 patients with primary HCC, those with other causes rather than HBV, HCV, or alcohol (*n* = 125), concurrent HBV and HCV (*n* = 7), or no available data regarding the cause of HCC (*n* = 8) were also excluded. In addition, 4 patients without available RNA sequence data for tumor tissue were excluded. Thus, 218 patients with primary HCC associated with HBV (HBV group, *n* = 95), HCV (HCV group, *n* = 47), or alcohol (alcohol group, *n* = 76) were finally enrolled in this retrospective study. HCC was pathologically diagnosed in all study subjects.

### Recruitment of demographic and laboratory data

Patient clinical information was downloaded from the TCGA database. Clinical data, such as, age, gender, race, causes of HCC, presence of LC, serum alpha-fetoprotein (AFP), albumin, prothrombin time (PT), bilirubin, Child-Turcotte-Pugh (CTP) classification, and tumor stage (American Joint Committee on Cancer, AJCC), were recruited. LC was pathologically defined based on the Ishak fibrosis scoring system for the surgically obtained peritumoral normal liver tissue [24, 25]. The AJCC tumor stages in the TCGA were initially recorded with the different versions from 4th to 7th because HCC tissues were obtained from the different times, and thus, it was newly adjusted to bring them into line with the AJCC 7th version in this study.

### Identification of alternative splicing events

RNA sequencing results were assessed to detect AS patterns in tumor tissues, and RNA-sequencing data was available for all 218 study subjects. Raw paired-end reads of RNA-seq Level 1 data were downloaded from the online Genomic Data Commons (GDC) Data portal [26]. Unaligned fastq files of study subjects were downloaded, and the reads were mapped to the reference genome (i.e., release 75, GRCH 37.75 based on the hg19 reference sequence) using STAR v2.5 [27]. All samples had unique mapping rates of over 80%. We trimmed all reads to same read lengths and ran MATs v3.2.5 [28] to identify AS events and quantify their expression level as a *percent spliced in* (PSI, ΔPSI or Δψ). PSI is the value estimated using proportions of exon-exon junction reads. All possible AS events, that is, exon skipping (ES), intron retention (IR), alternative 3′ splicing site (A3SS), alternative 5′ splicing site (A5SS), and mutually exclusive exons (MXE) were considered. The etiology groups were compared in pairs, i.e., HBV vs. HCV, HBV vs. Alcohol, and HCV vs. Alcohol. Although significantly differential expressed AS event is generally assessed with a ΔPSI > 10% (FDR of< 0.05) which means 10% difference is PSI between the groups [28]. We lowered a cutoff of a ΔPSI by 0.05 with an FDR-corrected *p*-value < 0.05 to include marginal signals in all comparisons.

### Protein-protein interaction analysis

Using gene sets from two different comparison groups, protein-protein interaction (PPI) networks were constructed using STRING *v*.10.5 dataset [29]. Only first gene interaction with a highest confidence of> 0.9 and without disconnected nodes was selected. PPI networks were visualized using Cytoscape v3.5.1 software [30].

### Canonical pathway and gene ontology analysis

Gene set enrichment analysis of the identified AS genes for the canonical pathway and gene ontology (GO) was performed using the consensus pathDB-human database (CPDB) [31]. We selected a significant pathway with an FDR < 0.05. Significant GO terms were yielded with FDR < 0.05. GO hierarchy level after limited from 2 to 5 categories to reduce redundant GO terms, and then potentially false positive GO terms were filtered out by using GO-Module [32].

### Statistical analysis

Patient baseline characteristics are presented as mean ± standard deviation, or frequencies. The significances of differences between categorical or continuous variables were determined using the *Chi*-square test, Fisher’s exact test, the Student’s *t* test, or ANOVA with post hocTukey’s b test, as appropriate. Two-tailed *P*-values of < 0.05 were considered statistically significant. In addition, to avoid false positives in multiple testing for alternative splicing analysis, FDR correction was performed. A corrected FDR of< 0.05 was considered to be statistically significant. The statistical analysis for clinical data was performed using SPSS v19.0 (SPSS Inc., Chicago, IL, USA). All statistical boxplots were generated using R v3.4.4.

## Results

### Clinical characteristics of patients

Patient’s clinical characteristics are summarized in Additional file 1: Table S1. Mean age in the HBV group was 54 years, which was significantly lower than those in HCV and alcohol groups, respectively (*P* < 0.05). The proportions of male in the three groups were similar (*p* = 0.92). In the HBV group, 88.4% of patients were of Asian origin, whereas in the HCV and alcohol groups, 63.8 and 63.2%, respectively, were of Caucasian origin. Serum albumin, total bilirubin, and PT levels were not significantly different between the groups (*p*-values for all> 0.05). The proportion of Ishak fibrosis-based cirrhosis was greater in the HCV and alcohol groups than in the HBV group (*p* < 0.05). AJCC (7th) stage IV was more common in the HBV group than in the HCV or alcohol groups (*p* < 0.05).

### Stratification of differentially expressed AS events in the three etiology groups

Identified AS events in the three groups are summarized in Additional file 1: Table S2. For the three intergroup comparisons HBV vs. HCV, HBV vs. alcohol, and HCV vs. alcohol (FDR < 0.05 and ΔPSI> 0.1), the numbers of differential AS events were 29 (22 genes), 15 (11 genes), and 5 (5 genes), respectively (Additional file 1: Table S2). As genes with AS events with 10% differences between the groups were not enough to further investigate functional relationships between three carcinogenesis, we included AS events with the marginal differences in all comparison by lowering to ΔPSI> 0.05 (FDR < 0.05), yielding differential AS event count of 133 (89 genes) from HBV vs. HCV, 93 (69 genes) from HBV vs. alcohol, and 29 (23 genes) from HCV vs. alcohol. The distribution of the different AS events (ES, IR, A3SS, A5SS, and MXE) in the three group comparisons is showed at Additional file 1: Figure S1. ES and MXE events were predominantly observed. We primarily conducted all analyses with the expanded gene sets including a marginal signal (ΔPSI> 0.05) and also confirmed whether any of their significant results can be consistent with the gene sets with greater different PSI value > 0.1.

**Fig. 2:**
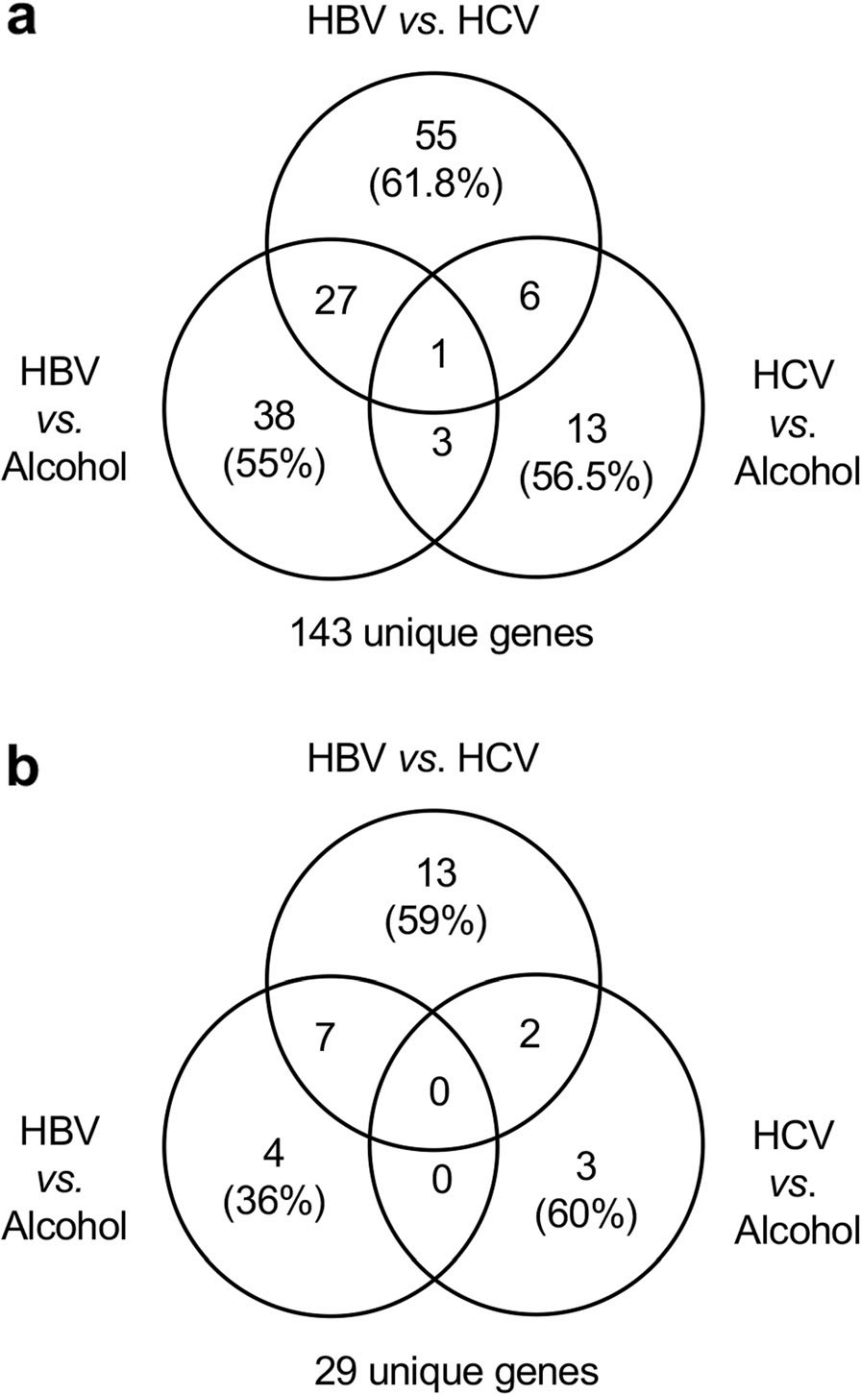
Identification of differentially expressed AS events in the three etiology groups. The total number of unique genes with identified AS events in three comparisons of HBV vs. HCV, HCV vs. alcohol, and HBV vs. alcohol were 143 (ΔPSI> 0.05) and 29 (ΔPSI> 0.1). **a** The numbers of unique gene exhibiting differential AS in the three comparisons (HBV vs. HCV, HBV vs. alcohol, and HCV vs. alcohol), were 55, 38, and 13, respectively. Moreover, 27, 6, and 3 genes were uniquely identified in the HBV, HCV, and alcohol groups, respectively. Only one gene was identified differentially in three different comparisons of each etiology group. **b** The numbers of unique differentially expressed AS genes observed for the three comparisons were 13, 3, and 4, respectively. Moreover, 7 and 2 genes were uniquely identified in the HBV and HCV groups, respectively. There was no gene differentially identified between each group comparison

As summarized in Venn-diagram (Fig. 2), the total number of unique genes with identified AS events in three comparisons were 143 (ΔPSI> 0.05) and 29 (ΔPSI> 0.1). Of these total 143 genes (FDR < 0.05 and ΔPSI> 0.05) (Fig. 2a), the numbers of unique gene exhibiting differential AS in each three comparisons (HBV vs. HCV, HBV vs. alcohol, and HCV vs. alcohol), were 55, 38, and 13, respectively. Only one gene was commonly shared in three different comparisons of each etiology group (Fig. 2a). For the 29 unique genes whose AS events has a greater differential expression (ΔPSI> 0.1) (Fig. 2b), the numbers of unique differentially expressed AS genes observed for the three comparisons were 13, 4, and 3, respectively. There was no gene commonly identified in three comparisons.

### Network and functional analysis of the three etiology groups comparison

**Fig. 3:**
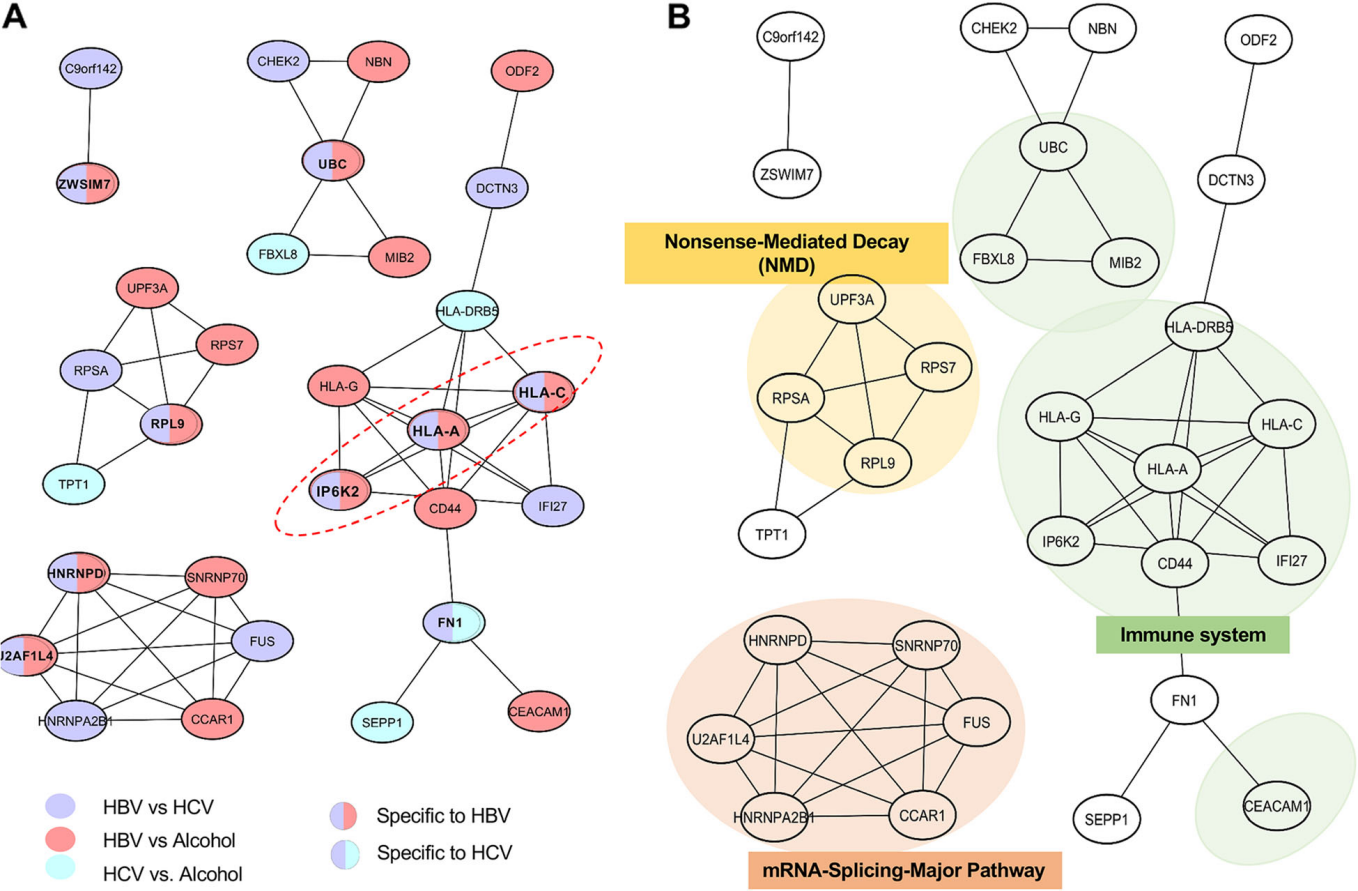
Network and functional analysis of the three etiology groups comparison. **a** Protein-protein interaction network (highest confidence> 0.9) of the 143 genes (FDR < 0.05 and ΔPSI> 0.05) that are differentially expressed for the three group comparisons HBV vs. HCV, HCV vs. alcohol, and HBV vs. alcohol. Colors represent comparisons of differential AS events between etiology groups (HBV vs Alcohol, HCV vs Alcohol, and HBV vs HCV). The red circled dash shows a PPI network of the 29 genes (FDR < 0.05 and ΔPSI> 0.1) that are differentially expressed for the three group comparisons HBV vs. HCV, HCV vs. alcohol, and HBV vs. alcohol. **b** Significant pathways obtained by enrichment analysis in the CPDB database for 143 genes; immune system (FDR = 0.013), mRNA splicing-major pathway (FDR = 0.007), and nonsense-mediated decay (FDR = 0.003). PSI, percent spliced in; HBV, hepatitis B virus; HCV, hepatitis C virus; PPI, protein-protein interaction; AS, alternative splicing

As mentioned in above, of the 143 genes (FDR < 0.05, ΔPSI> 0.05) identified by group comparisons of HBV vs. HCV, HCV vs. alcohol, and HBV vs. alcohol, 106 (25%) were uniquely spliced in only one of each comparison, and 36 (75%) were shared in at least two comparisons. In order to investigate how functionally these differentially or commonly spliced genes are associated with each risk factor in HCC development at the system level, we conducted network analysis and functional annotation as described in Methods. Among 143 genes, 30 genes were identified to have a known PPI relationship according to the STRING database and structured on the network (Fig. 3a). In the network, there were eight HBV-group specific AS genes (both purple and red color) and one HCV-group specific AS gene (both purple and blue color), but no alcohol-specific AS gene. Furthermore, seven (purple color only), ten (red color only), and four genes (blue color only) showed differential AS events in comparison of HBV vs. HCV, HBV vs. alcohol, and HCV vs. alcohol, respectively (Fig. 3a).

In addition, we further conducted functional analysis over the canonical pathway and the GO terms for the 143 genes. The significantly identified known canonical pathways (Additional file 1: Figure S2) were overlaid in the PPI network to topologically evaluate the functional distance among the etiology-specific or common AS events (Fig. 3b). Interestingly, we identified three canonical pathways enriched in the AS genes, as a subnetwork, immune system (eleven genes*,* FDR = 0.013), mRNA splicing-major pathway (six genes*,* FDR = 0.007), and nonsense-mediated decay (NMD) (four genes*,* FDR = 0.021). Consistently, we also found several significant GO terms as similar to the identified canonical pathways (Additional file 1: Figure S3) including innate immune response (GO: 0045087, FDR = 0.027), response to type I interferon (GO: 0034340, FDR = 0.004), interferon-gamma-mediated signaling pathway (GO: 0060333, FDR = 0.006), and cellular response to cytokine stimulus (GO: 0071345, FDR = 0.029).

In terms of etiology specific AS gene, four of the eight AS genes specific to HBV-group (both purple and red color) were commonly implicated in the immune system pathway, two in the mRNA-splicing Major pathway, and one in the NMD pathway. Especially, of the eleven genes in the immune system pathway, seven (*HLA-A, HLA-C, HLA-DRB5, IP6K2, IFI27, HLA-G,* and *CD44*) were commonly associated with interferon signaling (i.e., interferon gamma signaling, FDR = 0.001; interferon alpha/beta signaling, FDR = 0.001) as determined by enrichment analysis in the CPDB database. For example, *HLA-A* is known to be in the Immune system pathway (Fig. 3b) to activate cytotoxic T-cell signaling pathway in cancer. In addition, *CD44* and *HLA-DRB5* which are directly interacted with HLA-A are known to be implicated by interferon signals in different cancers [33–35]. For example, CD44 expression can be decreased by interferon gamma (IFNg) in ovarian carcinoma cells [35]. Moreover, HLA-DRB5 is up-regulated by interferon alpha2 (IFN) in Polycythemia Vera of blood cancer [36]. These genes play a significant role in immune system via interferon signaling.

Of seven genes (purple color only) with differential AS events between the HBV vs. HCV groups, one was specifically implicated in the Immune system pathway, two in the mRNA-splicing Major pathway, and one in the NMD pathway. Of ten genes (red color only) differentially spliced in comparison of the HBV vs. alcohol groups, one was specifically implicated in the Immune system pathway, two in the mRNA-splicing Major pathway, and one in the NMD pathway. In addition, of four genes (blue color only) spliced in comparison of the HCV vs. alcohol groups, two were implicated in the Immune system pathway.

We selected 29 unique genes with stronger differential values (ΔPSI> 0.1) between the groups, and of those 29, three genes (i.e., *HLA-A*, *HLA-C,* and *IP6K2)* were found in the PPI network (Fig. 3a; red circle). These genes commonly showed strong differential AS event (ΔPSI> 0.1) and were specifically implicated in the immune system pathway as HBV-specific AS genes (Fig. 3b). For these genes, case studies were performed to evaluate the functional or clinical impact of AS event, *HLA-A*, *HLA-C*, and *IP6K2*.

### Case study 1: functional impact of AS event in HLA-A

**Fig. 4:**
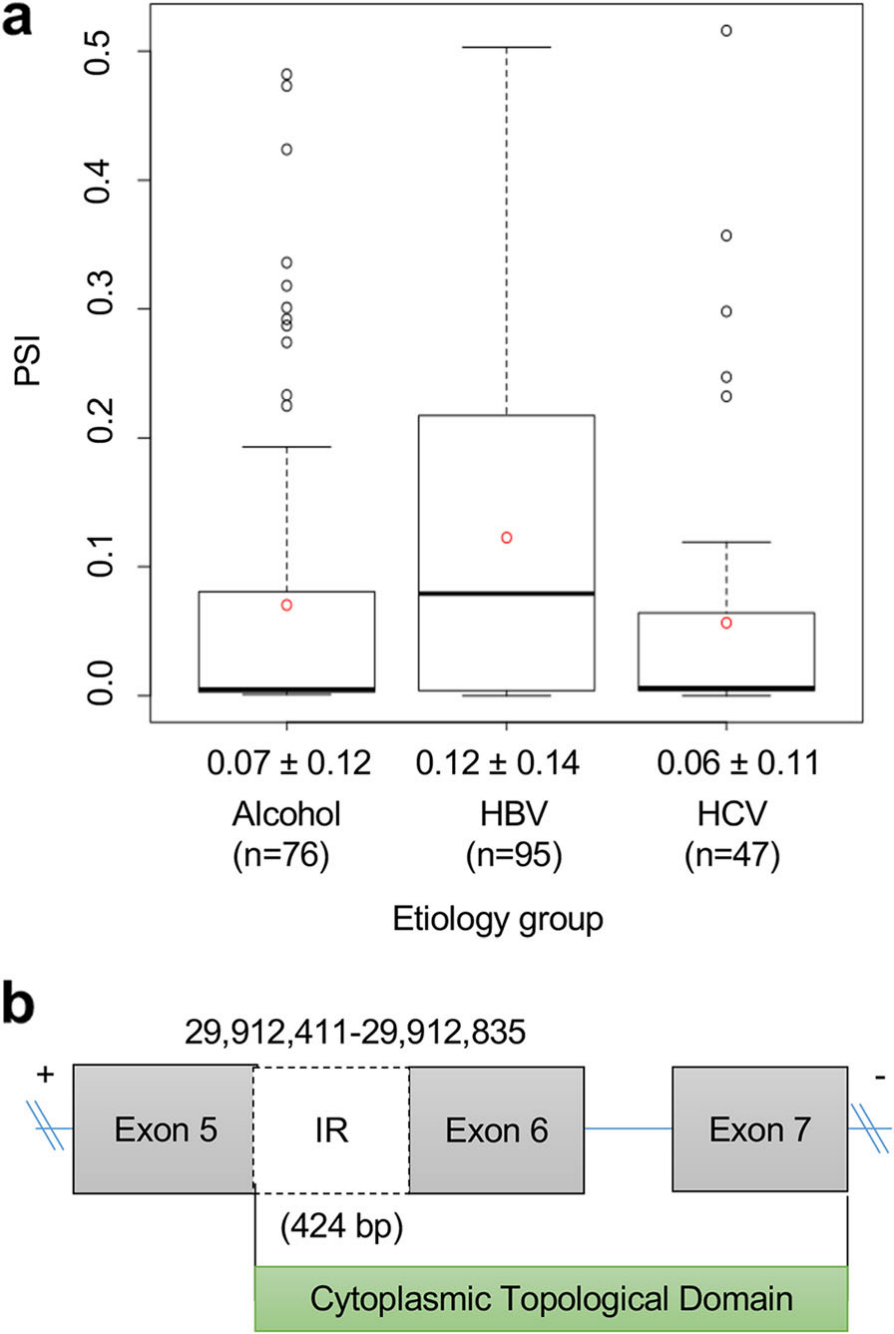
Alternative splicing event of *HLA-A.* **a** Schematic of an IR event between exon 5 and exon 6 on the *HLA-A* transcript. **b** PSI level of IR exon 5 of *HLA-A* in the three different etiology groups (HBV, HCV, and alcohol groups)

Human leukocyte antigen (HLA) is a human specific major histocompatibility complex (MHC) antigen and consists of HLA class I and II. HLA plays a pivotal role in the human immune system, and its expression is associated with malignant transformation in human body [37]. Our study found that intron 5 of *HLA-A*, which has ten exons, tends to be more frequently retained in the HBV group (mean PSI = 0.12) compared to the HCV (mean PSI = 0.06) and alcohol groups (mean PSI = 0.07) (Fig. 4a). Interestingly, this retained intron 5 spanning exons 5 and 6 potentially introduces a new protein sequence and affects the protein domain, “cytoplasmic topological domain”, which is encoded by exons 5, 6, and 7 (Fig. 4b). Furthermore, the length of retained intron 5 is 424 bp, which introduces the frame shift, and therefore, produces different or truncated protein product.

### Case study 2: functional impact of AS event in HLA-C

**Fig. 5:**
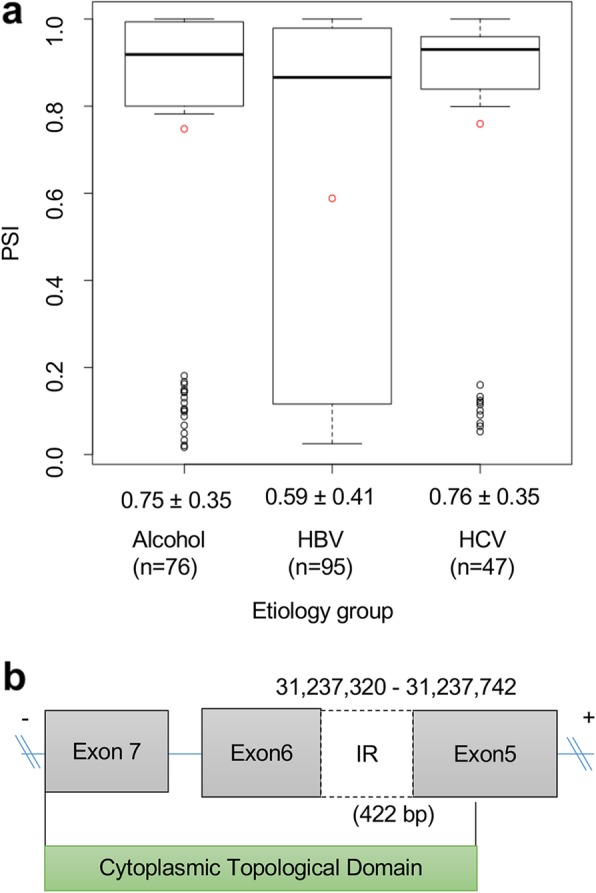
Alternative splicing event of *HLA-C.* **a** Schematic of an IR event between exon 5 and exon 6 on the *HLA-C* transcript, **b** PSI level of IR exon 5 of *HLA-C* in the three different etiology groups (HBV, HCV, and alcohol groups). HLA, human leukocyte antigen; IR, intron retention; PSI, percent spliced in; HBV, hepatitis B virus; HCV, hepatitis C virus

*HLA-C* is also an HLA-class I gene, known as a killer cell immunoglobulin-like receptor and associated with HBV-related HCC development [38]. We identified that intron 5 of *HLA-C,* which has eight exons, tends to be less frequently retained in the HBV group (mean PSI = 0.59) compared to the HCV (mean PSI = 0.76) and alcohol groups (mean PSI = 0.75) (Fig. 5a). Interestingly, this retained intron 5 spanning exons 5 and 6 potentially introduces a new protein sequence and affects the protein domain, “cytoplasmic topological domain”, which is encoded by exons 5, 6, and 7 (Fig. 5b). Furthermore, the length of retained intron 5 is 422 bp, which produces the frame shift, leading to different or truncated protein product.

### Case study 3: clinical implication of AS event in IP6K2

**Fig. 6:**
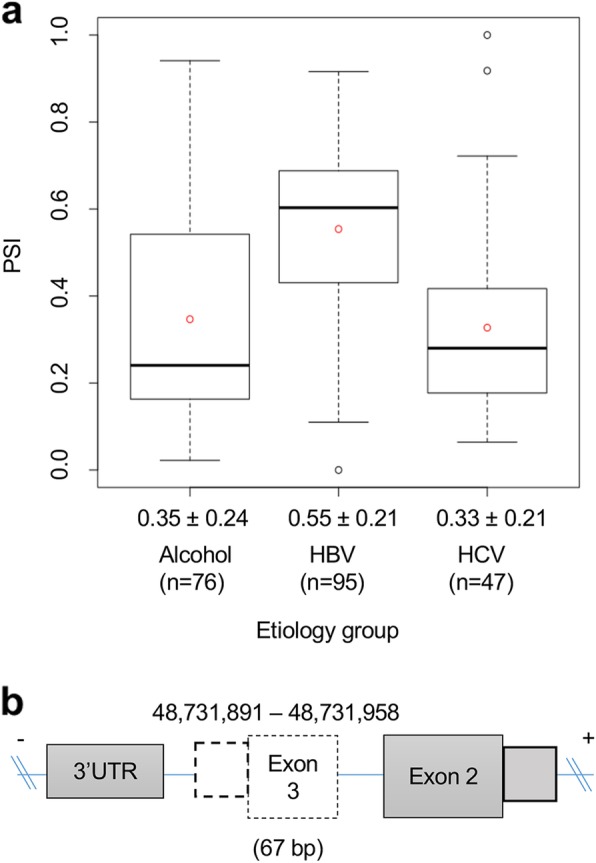
Alternative splicing events of *IP6K2.* **a** Schematic of skipping exon 3 on the *IP6K*2 transcript, **b** PSI level of ES of *IP6K*2 in the three different etiology groups (HBV, HCV, and Alcohol groups). IP6K2, inositol hexakisphosphate kinase 2; ES, exon skipping; PSI, percent spliced in; HBV, hepatitis B virus; HCV, hepatitis C virus

*Inositol hexakisphosphate kinase 2* (*IP6K2*), which has six exons in the canonical transcript, is known to be associated with cancer cell migration, invasion, and tumor metastasis, but it has not been established how this gene affects HCC development related with the three etiologies [39]. We found exon 3 (based on ENST00000432678) of *IP6K2* tends to be more frequently skipped in the HBV group (mean PSI = 0.55) compared to the HCV (mean PSI = 0.35) and alcohol groups (mean PSI = 0.33) (Fig. 6a). Although the skipped exon 3 is not part of domain coding regions, it may potentially affect a post-transcription regulation due to the frameshift, leading to the longer 3′ UTR which might be more chance to be regulated by miRNA (Fig. 6b).

### Subgroup analysis of differential AS events in Caucasian and Asian patients

HCC incidences are population dependent [40, 41]. For example, Asians or Africans has higher incidence in HCC compared to Caucasians [40, 41]. Thus, we assessed whether our identified AS are solely etiology depended or ethnicity is another confounder. We sub-grouped the study subjects into Caucasians (*n* = 85) and Asians (*n* = 114) groups according to the TCGA clinical data (Additional file 1: Table S3) and performed the *t*-test for group comparison of PSI value to identify differential AS genes between the groups. The numbers of differential AS events between the two populations were 33 (FDR < 0.05 with ΔPSI> 0.1) corresponding to 25 genes and 363 (FDR < 0.05 with ΔPSI > 0.05) corresponding to 198 genes, respectively (Additional file 1: Table S4). When the first PPI network were constructed for the 198 unique genes (FDR < 0.05, ΔPSI> 0.05), 45 genes were found to construct the network. To identify the genes associated with etiologic differences specifically, but with racial differences, we superimposed the PPI network of these 45 genes on the top of the PPI network, which consisted of 30 genes and were identified comparing the three etiology groups (Additional file 1: Figure S4). Of the 45 genes showing differential AS events between the two racial groups, 15 genes were found to be differentially AS events between the etiologic groups. Thus, of these 30 genes showing differential AS events between the etiologic groups, 15 (white color) were found not to be affected by the racial difference.

## Discussion

In this genome-wide analysis of AS events among three etiologies, we found 133, 93, and 29 differential AS events (FDR < 0.05, PSI > Δ0.05) were identified in the group comparisons of HBV vs. HCV, HBV vs. Alcohol, and HCV vs. Alcohol, respectively. For corresponded 143 AS genes, we conducted functional annotation and found three enriched canonical pathways (i.e., immune system, mRNA splicing-major pathway, and NMD) implicated in HCC development. The present study is unique because to our knowledge, it is the first study that investigated genome-wide screening of AS events and their implication in the three key risk factors in HCC. Moreover, this study showed some of the differential AS genes between the groups were commonly implicated in one of the three canonical pathways.

Generally, HCC occurs after several years of hepatocyte inflammation [42] caused by chronic HBV or HCV infection or alcoholic cirrhosis, but the molecular mechanism responsible for HCC development differ slightly between these etiologies [2–4]. Recent advances in NGS have provided significant insights of the molecular mechanism underlying HCC carcinogenesis [18–20]. Nonetheless, the reasons why the mechanisms associated with these three etiologies are different, especially at the transcriptome level, such as AS, are not well known. In a previous study, it was reported AS patterns of transcripts are different between HBV- and HCV-associated HCCs [22]. However, alcohol-associated HCC was not included, and mixed HCCs were also enrolled in the study [22]. Therefore, in the present study, we conducted the comprehensive and genome-wide finding of differential AS events that exist among these three etiology groups using only primary HCC.

In the present study, we identified the *HLA-A*, *HLA-C*, and *IP6K2* were specifically associated with the differential AS events whose AS events were HBV specific. HLA is a human specific major histocompatibility complex (MHC) antigen, and consists of HLA class I and II, which plays a pivotal role in the human immune system, and its expression is associated with malignant transformation in human body [37]. Indeed, downregulation or loss of HLA-A antigen has been reported to occur in 16–50% of malignant tumors [37], and this antigen is known to act as a regulatory and transcription factor to maintain HLA-A heavy chain expression [43]. Reduced HLA-A expression allows tumor cells to escape from immune system, especially cytotoxic T-cell signaling pathway [44], leading to tumor progression and poor outcomes in different tumors, such as breast, colorectal, ovarian cancer [45–47]. On the other hand, HCC is frequently associated with upregulation of HLA-A expression [48]. However, available information cannot explain the exact mechanism implicated in this phenotypic change. Although it was reported in a previous study that two transcription factors (p65 and interferon regulatory factor 1) are required for HLA-A upregulation in HCC, it did not demonstrate a difference between HBV- and HCV-associated HCCs with respect to HLA-A upregulation [49]. This may be result by that the majority (87%) of HCC was HBV-associated, and protein expressions were assessed by Western blotting in the previous study. However, in the present study, we used mRNA sequencing to identify molecular difference at the transcript level, and interestingly, we identified the intron 5 of *HLA-A* was more frequently retained in HBV-associated HCC than in HCV- or alcohol-associated HCC. More importantly, a part of cytoplasmic topological domain may be affected by the retained intron (Fig. 4a). As described in Results, the length of retained intron, 424 bp, is not multiple by three, and thus, it uses the different frame for translation to protein sequences. Even if HLA-A expression was reported to increase in HCC as mentioned above, specific splicing pattern of *HLA-A* in HBV-associated HCC may affect its function by using different frame for translation from other cancer types. This finding suggests intron 5 might regulate HBV-associated HCC, and immune escape to HLA-A by tumor cell in this tumor might occur via intron retention. With regard to the lack of a significant difference between HCV- and alcohol-associated HCCs with regard to IR events, it implies that HLA-A may be dysregulated by splicing in only HBV-associated HCC and its IR may be an important factor underlying molecular mechanism specific to HBV carcinogenesis.

*HLA-C* is also one of HLA-class I genes, and *HLA-C* has been known as a killer cell immunoglobulin-like receptors and to be associated with HBV-associated HCC development [38]. In the present study, we sought to identify differential AS events and corresponding genes in HBV-, HCV-, and alcohol-associated HCCs, and found intron 5 of *HLA-C* had a lower PSI level specifically in HBV-associated HCC than in the other groups. Moreover, similar to *HLA-A,* we observed cytoplasmic topological domain effect on IR events of exon 5. Interestingly, unlike *HLA-A*, IR events of exon 5 occurred less frequently in HBV-associated HCC, which suggests that *HLA-A* and *-C* may have effects on the development of HBV-associated HCC via slightly different immune mechanisms. Given that different AS event are associated with the development of these HCCs, our findings might be helpful for establishing immune-mediated therapeutic concepts for HBV-, HCV-, and alcohol-associated HCCs.

*IP6K2* is known to be associated with cancer cell migration, invasion, and tumor metastasis [39]. However, in terms of the development of HBV-, HCV-, and alcohol-associated HCCs, it has not been well known how this gene affects HCC development. Interestingly, in the present study, we found alcohol- and HCV-associated HCCs were associated more frequently with skipping of exon 3 (based on ENST00000432678) than HBV-associated HCC. Skipping of this exon also alters the reading frame of downstream exons, potentially marking its transcript as an NMD candidate. This observation suggests that the differential exon skipping pattern of *IP6K2* is associated with the different characteristics, such as tumor metastasis, of these HCCs. Based on the baseline characteristics of HCC patients in the present study, metastatic tumors (TNM stage IV) were more frequent in HBV-associated HCC than in HCCs with the other two etiologies. Because the present study was conducted using data on tumor tissues obtained from HCC patients treated by surgical resection, the number of metastatic HCCs was inevitably small. Therefore, we suggest a large scald study that includes metastatic HCCs be conducted. Intron retention often causes a premature termination codon, introducing a frame shift and leading to NMD process. As our three genes (*HLA-A*, *HLA-C*, and *IP6K2*) may be candidates for NMD, we investigated whether the effect of NMD regulation on their gene-level expression would be evident among the three etiologies. We found that *HLA-A* expression was lower in HBV-associated HCC than in the other two groups (Additional file 1: Figure S5). We also observed higher intron retention for HLA-A in HBV-associated HCC. Taken together, these finding may imply that increased intron retention contributes to the lower overall gene expression in HBV-associated HCC via the mechanism of NMD.

In the present study, three biological pathways were found for the 143 genes identified by comparing the HBV, HCV, and alcohol groups, and 30 genes constitute the PPI network. First, 11 (36.7%) of 30 genes were associated with the immune system pathway. Although HCC patients exhibit different patterns of the immune system alterations, dysregulation of the immune system is known to play an important role in the pathogenesis of HCC, especially in HBV or HCV-associated HCC [50]. Interestingly, in the present study, seven of the eleven genes in the immune system pathway were commonly associated with interferon signaling by enrichment analysis in the CPDB database. For example, *HLA-A* directly interact with *CD44* and *HLA-DRB5* which are known to be related with interferon signaling in carcinogenesis. Given that interferons are closely associated with anti-tumor immune responses, these genes may be potential targets for immune-medicated anti-tumor therapy in these HCCs. Moreover, cellular immunotherapy has the potential to improve treatment outcomes in HCC, and thus, our data might be found useful by those investigating immune-based therapeutic approaches to HBV-, HCV, or alcohol-associated HCCs. Second, in terms of the mRNA splicing major pathway, profiling of molecular changes in HCC has revealed multiple targets [51]. However, little is known of RNA splicing in HCCs with the three different etiologies (HBV, HCV, and alcohol). Although HBV-associated HCC was compared with HCV-associated HCC in a recent study, genes that make up the splicing pathway were not included, whereas in the present study, we identified an mRNA splicing pathway containing 6 genes. Third, the NMD pathway is present in all eukaryotes, and has a type of surveillance function because it reduces errors in gene expression by removing mRNA transcripts containing premature stop codons [52]. In particular, up-frame shift (*UPF*) is known to constitute the conserved core of the NMD pathway, and to consist of *UPF1, UPF2* and *UPF3* (*UPF3A* and *UPF3B* in humans) [53]. In the present study, *UPF3A* was found to constitute the NMD pathway, and is known to be part of the exon-exon junction complex (EJC) bound to mRNA after AS [53]. Furthermore, it was reported *UPF1* acts a tumor suppressive gene in HCC [54], but, the role played by *UPF3A*, in the pathogenesis of HCC, has not been well studied. The identification of these three pathways suggests AS plays important roles in the carcinogenesis of HBV-, HCV-, and alcohol-related HCC. In order to verify the effect of the AS event in *UPF3A* on NMD, we investigated the mRNA levels of NMD target genes. There were eight target genes that could be affected by *UPF3A* via the NMD process [55]. Among them, we found that *NR3C2* tends to have lower expression in HBV-associated HCC than in the other two groups (Additional file 1: Figure S6). Our PPI results (Additional file 1: Figure S4) indicate that *UPF3A* interacts with *RPL9*, which was identified as an HBV-specific AS gene in this study. That is, this may suggest that the splicing of *RPL9* might alter the *UPF3A*-mediated NMD of *NR3C2*. In addition, NR3C2 was recently revealed to be a potential tumor-suppressor gene in pancreatic cancer. Thus, the lower expression of *UPF3A* in HBV-associated HCC may play a role in HCC progression [56].

The gene *CD46* (membrane cofactor protein, MCP) was common to three different comparisons among etiology groups (FDR < 0.05, and PSI > 0.05). This gene contributes to the complement regulatory protein and is specifically known for being part of the membrane-bound complement –regulatory proteins (mCRPs). Many studies have shown that *CD46* expression in HCC tissue is significantly higher than in normal tissue [57–59]. Also, *CD46* is known to have alternative splicing (exons 7–9) [60, 61], and we observed a mutually exclusive exon 8 in the HBV vs. HCV comparison and an alternative 5′ splice site (exon 6) in both HBV vs. Alcohol and HCV vs. Alcohol group comparisons. That is, *CD46* may be a candidate molecular biomarker for HCC. A list including the frequently altered genes that are common to all etiologies is given in Additional file 1: Table S5. The etiology of HCC exhibits ethnic differences. In Asians, 80–90% of HCC cases are associated with HBV or HCV, whereas in the USA, only 20–50% of the population is so associated [40, 41]. which may suggest HCC development in Caucasian may be less dependent on HBV or HCV. In the present study, we also found these etiologic differences between Caucasians and Asians, which introduces the possibility that mechanisms of HCC carcinogenesis ethnically dependent may be present, and ethnic difference may be a confounder for differential AS events between the etiologic groups. Accordingly, we performed subgroup analysis on AS events in Caucasians and Asians, and found 15 genes (FDR < 0.05, ΔPSI> 0.05) were etiologically specific, but not affected by the ethnicity. Given few reports have been issued on AS mechanisms at transcriptome level associated with the correlation between HCC development and etiology, we believe these results provide useful information regarding the relations between genomic landscapes of HCC occurrence in these etiologic groups, not affected by ethnicity.

Another factor affecting alternative splicing is a splicing regulatory element (SRE). It is a hexametric motif as a cis-regulatory element (i.e., exonic splicing enhancer, exonic splicing silencer, intronic splicing enhancer, and intronic splicing silencer) [62], and it often regulate alternative splicing. The existence of these SREs around exons and intron could be another important factor contributing to the differential splicing level across etiologies. Most of all, if genetic variant occurs within the SRE, the splicing would be further affected [63, 64].

Several limitations of the present study require consideration. First, inherent selection bias could not be avoided due to its retrospective design. Second, the results of this study were not validated using patient blood samples. Thus, validation studies using patient serum and tumor tissue samples are required. However, given the high cost of RNA sequencing, the results of the present study provide an important basis for future research. Third, due to the small number of available mortality data for HCC patients, we could not address the association between specific genes and patient survival. A well-designed prospective study is needed to investigate such an association.

## Conclusions

In conclusion, in this genome-wide study, we found AS of *HLA-A*, *HLA-C,* and *IP6K2* were differently expressed in HBV-, HCV-, and alcohol-associated HCC. In addition, we identified three different significant pathways (immune system mRNA splicing-major pathway, and nonsense-mediated decay) and their corresponding genes were associated with the carcinogenesis of these three HCC types (FDR < 0.05). Moreover, some of the specifically identified genes in the inter-group comparisons between the three etiology groups were commonly implicated in one of the three canonical pathways. Also, we found that 15 genes showed specifically differential AS events between the etiology groups, not affected by ethnic differences for HCC. We believe this investigation of transcriptional differences between HBV-, HCV-, and alcohol-associated HCC provides insight at a molecular level of the functional relationships between risk factors of HCC.

## Supplementary information

**Additional file 1: Figure S1.** Count of alternative splicing events. (A) Gene set were selected with FDR < 0.05 and PSI > 0.1. (B) Gene set were selected with FDR < 0.05 and PSI > 0.05.**Figure S2.** Canonical pathways enriched in 143 alternatively spliced genes. Red line indicates the cutoff FDR value, q < 0.05. **Figure S3.** GO terms enriched in 143 alternatively spliced genes. GO Cellular Component (CC), Biological Process (BP), and Molecular Function (MF) terms were considered significant at q < 0.05 (red line). Potential false positive GO terms were filtered. GO, gene ontology. **Figure S4.** PPI not affected by ethnic differences. Protein-protein interaction (PPI) network analysis was conducted for 198 genes (FDR < 0.05, ΔPSI > 0.05) differentially associated with AS events in Caucasians and Asians. Of the 30 networked genes from the three etiology group comparisons (Fig. 3), 15 genes (colored) were affected by ethnic differences and 15 genes (non-colored) were not affected by ethnicity. **Figure S5.** Boxplots for distributions of gene expression among three etiologic groups for our three case studies. **Figure S6.** Boxplots for distributions of gene expression among three etiologic groups. All of eight genes are reported to be affected by *UPF3A* via NMD process. **Table S1.** Baseline clinical characteristics of study subjects. **Table S2.** Identification of alternative splice events in the three pairwise comparisons of HBV vs. HCV, HCV vs. alcohol, and HBV vs. alcohol. **Table S3.** The demographic summary of Caucasian and Asian population. **Table S4.** Identification of alternative splice events in comparison of Caucasian and Asian population. **Table S5.** List of frequently altered genes by etiology.

## Data Availability

We gratefully acknowledge the TCGA Consortium, and all its members for the TCGA Project initiative, for providing a sample, tissues, data processing and making data and results available. The results published here are in whole or part based upon data generated by The Cancer Genome Atlas pilot project established by the NCI and NHGRI. Information about TCGA and the investigators and institutions that constitute the TCGA research network was published [65].

## Abbreviations

A3SS: Alternative 3′ splicing site
A5SS: Alternative 5′ splicing site
AFP: Alpha-fetoprotein
AJCC: American Joint Committee on Cancer
AS: Alternative splicing
CPDB: Consensus PathDB-human database
CTP: Child-Turcotte-Pugh
ES: Exon skipping
GDC: Genomic Data Commons
GO: Gene ontology
HBV: Hepatitis B virus
HCC: Hepatocellular carcinoma (HCC)
HCV: Hepatitis C virus
HLA: Human leukocyte antigens
IP6K2: Inositol hexakisphosphate kinase-2
IR: Intron retention
LC: Liver cirrhosis
MXE: Mutually exclusive exons
NGS: Next-generation sequencing
NMD: Nonsense-mediated decay
PPI: Protein-protein interaction
PSI: Percent spliced in
PT: Prothrombin time
RFs: Risk factors
TCGA: The Cancer Genome Atlas
UPF: Up-frameshift
