# Supplementary material for: Differential alternative splicing regulation among hepatocellular carcinoma with different risk factors
Source: BMC Med Genomics. 2019 Dec 20;12(Suppl 8):175. doi: 10.1186/s12920-019-0635-z (PMC6923823; doi:10.1186/s12920-019-0635-z)
Supplement: Supplementary file 1 — Additional file 1: Figure S1. Count of alternative splicing events. (A) Gene set were selected with FDR < 0.05 and PSI > 0.1. (B) Gene set were selected with FDR < 0.05 and PSI > 0.05.Figure S2. Canonical pathways enriched in 143 alternatively spliced genes. Red line indicates the cutoff FDR value, q < 0.05. Figure S3. GO terms enriched in 143 alternatively spliced genes. GO Cellular Component (CC), Biological Process (BP), and Molecular Function (MF) terms were considered significant at q < 0.05 (red line). Potential false positive GO terms were filtered. GO, gene ontology. Figure S4. PPI not affected by ethnic differences. Protein-protein interaction (PPI) network analysis was conducted for 198 genes (FDR < 0.05, ΔPSI > 0.05) differentially associated with AS events in Caucasians and Asians. Of the 30 networked genes from the three etiology group comparisons (Fig. 3), 15 genes (colored) were affected by ethnic differences and 15 genes (non-colored) were not affected by ethnicity. Figure S5. Boxplots for distributions of gene expression among three etiologic groups for our three case studies. Figure S6. Boxplots for distributions of gene expression among three etiologic groups. All of eight genes are reported to be affected by UPF3A via NMD process. Table S1. Baseline clinical characteristics of study subjects. Table S2. Identification of alternative splice events in the three pairwise comparisons of HBV vs. HCV, HCV vs. alcohol, and HBV vs. alcohol. Table S3. The demographic summary of Caucasian and Asian population. Table S4. Identification of alternative splice events in comparison of Caucasian and Asian population. Table S5. List of frequently altered genes by etiology. [file 12920_2019_635_MOESM1_ESM.pdf]

Supplementary Information

Differential alternative splicing regulation among hepatocellular carcinoma with different risk factors

Young-Joo Jin[1,2#], Seyoun Byun[1#], Seonggyun Han[1], John Chamberlin[1], Dongwook Kim[1], Min Jung Kim[1,3], and Younghee Lee[1,4*]

[1]Department of Biomedical Informatics, University of Utah School of Medicine, Salt Lake City, Utah, USA;
[2]Division of Gastroenterology, Department of Internal Medicine, Inha University School of Medicine, Incheon, South Korea;
[3]Pharmacy program, Massachusetts College of Pharmacy and Health Sciences, Worcester Massachusetts
[4]Huntsman Cancer Institute, University of Utah School of Medicine, Salt Lake City, Utah, USA

[#]These two authors (Young-Joo Jin and Seyoun Byun) are co-first authors.

Corresponding Authors: Younghee Lee, Ph.D.

Corresponding authors' address: Younghee Lee
Department of Biomedical Informatics
University of Utah School of Medicine
Salt Lake City, Utah, USA

Corresponding authors' e-mail address: younghee.lee@utah.edu

Additional file 1 (.pdf):

Figure S1. Count of alternative splicing events. (A) Gene set were selected with FDR < 0.05 and PSI > 0.1. (B) Gene set were selected with FDR < 0.05 and PSI > 0.05.

Figure S2. Canonical pathways enriched in 143 alternatively spliced genes. Red line indicates the cutoff FDR value, q < 0.05.

Figure S3. GO terms enriched in 143 alternatively spliced genes. GO Cellular Component (CC), Biological Process (BP), and Molecular Function (MF) terms were considered significant at q < 0.05 (red line). Potential false positive GO terms were filtered. GO, gene ontology.

Figure S4. PPI not affected by ethnic differences. Protein-protein interaction (PPI) network analysis was conducted for 198 genes (FDR <0.05, ΔPSI >0.05) differentially associated with AS events in Caucasians and Asians. Of the 30 networked genes from the three etiology group comparisons (Figure 3), 15 genes (colored) were affected by ethnic differences and 15 genes (non-colored) were not affected by ethnicity.

Figure S5. Boxplots for distributions of gene expression among three etiologic groups for our three case studies.

Figure S6. Boxplots for distributions of gene expression among three etiologic groups. All of eight genes are reported to be affected by UPF3A via NMD process.

Table S1. Baseline clinical characteristics of study subjects.

Table S2. Identification of alternative splice events in the three pairwise comparisons of HBV vs. HCV, HCV vs. alcohol, and HBV vs. alcohol.

Table S3. The demographic summary of Caucasian and Asian population.

Table S4. Identification of alternative splice events in comparison of Caucasian and Asian population.

Table S5. List of frequently altered genes by etiology

(A)

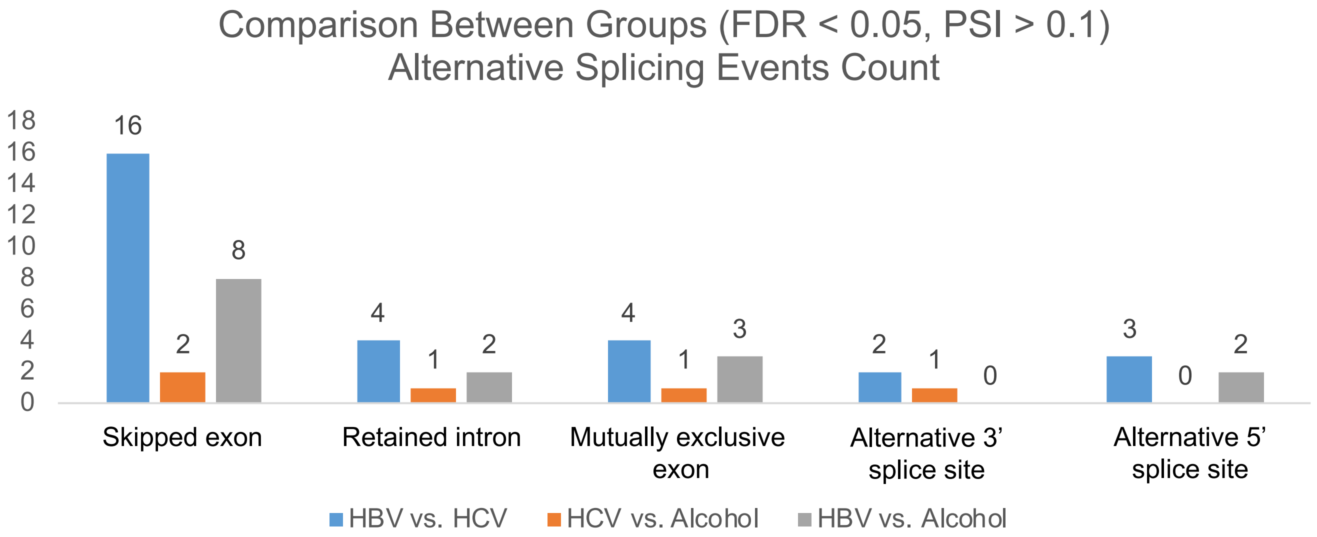

(B)

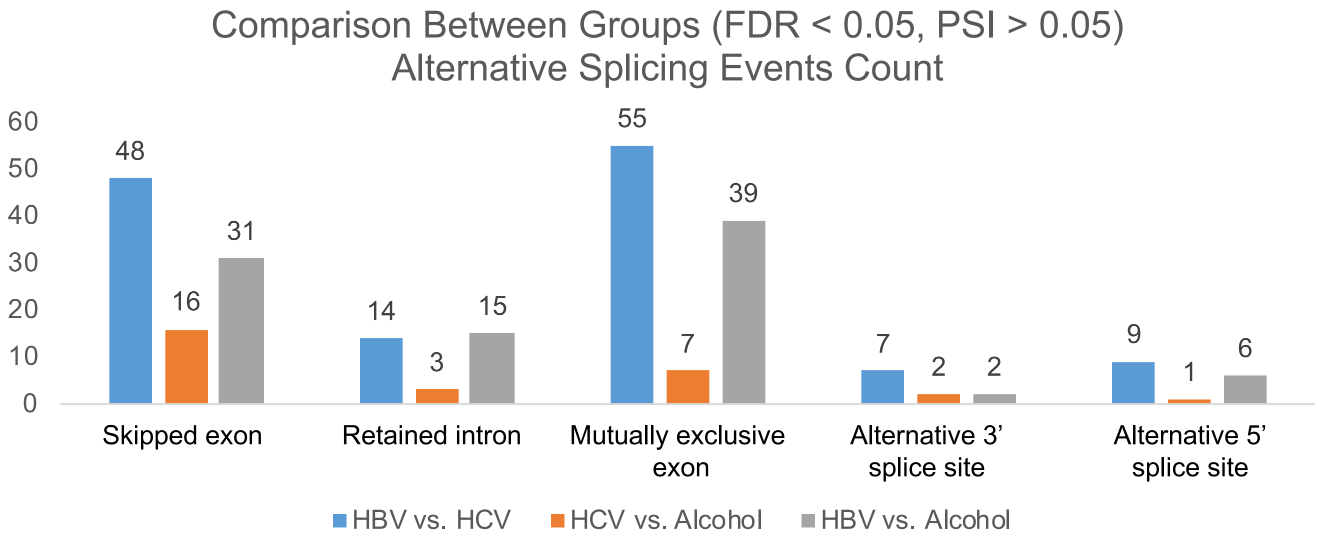

Figure S1. Count of alternative splicing events. (A) Gene set were selected with FDR < 0.05 and PSI > 0.1. (B) Gene set were selected with FDR < 0.05 and PSI > 0.05.

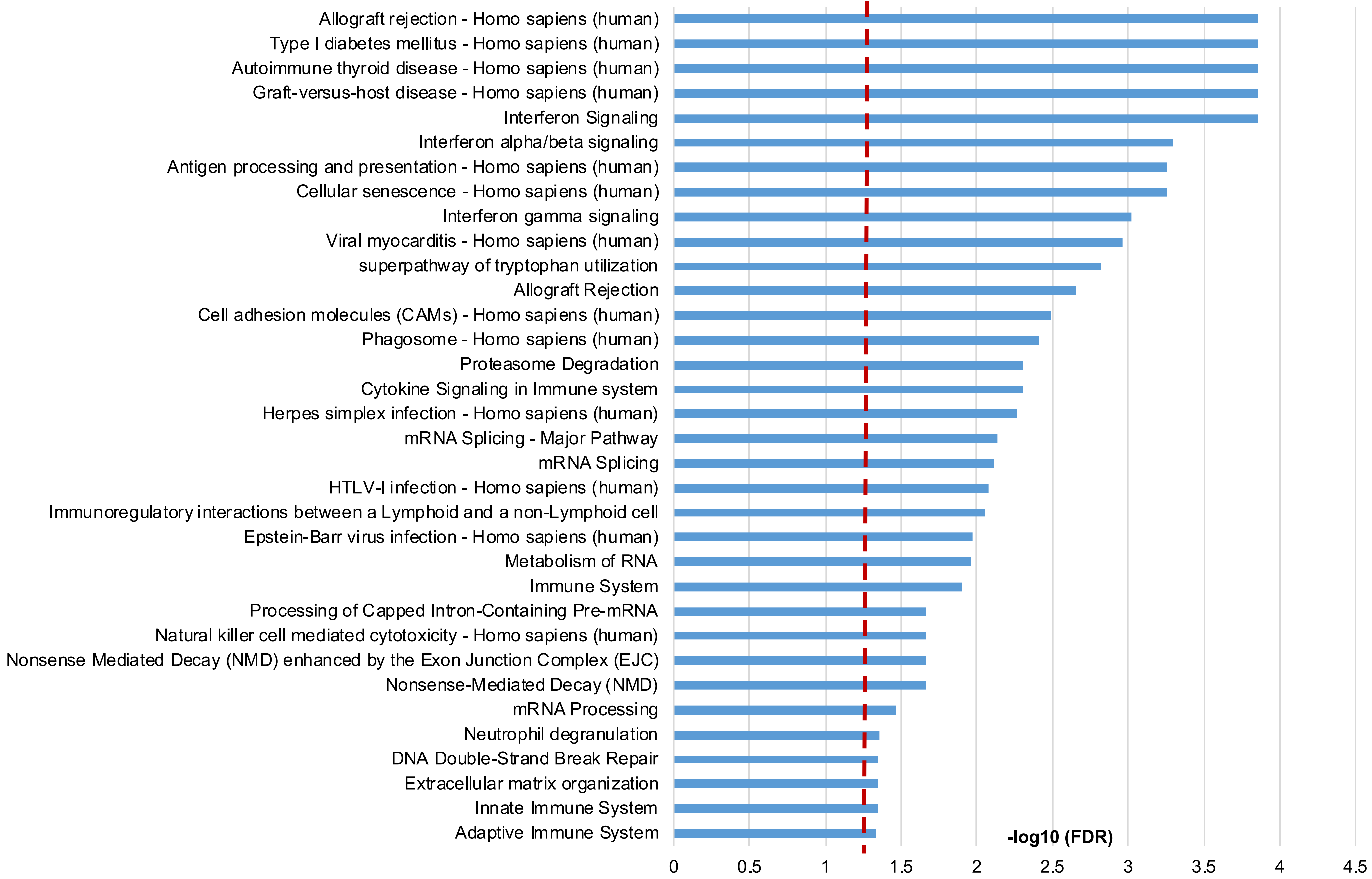

Figure S2. Canonical pathways enriched in 143 alternatively spliced genes. Red line indicates the cutoff FDR value, q < 0.05.

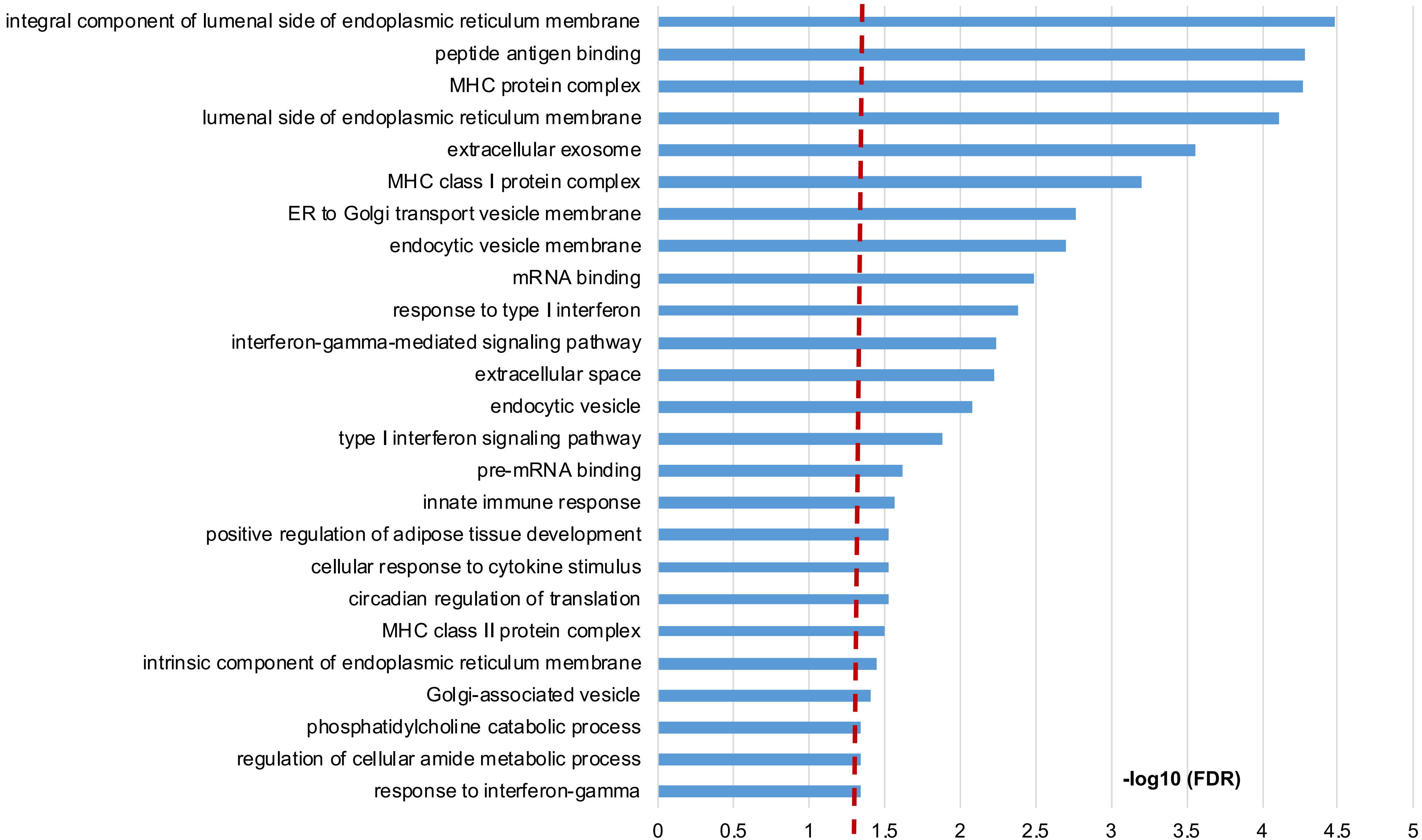

Figure S3. GO terms enriched in 143 alternatively spliced genes. GO Cellular Component (CC), Biological Process (BP), and Molecular Function (MF) terms were considered significant at $q < 0.05$ (red line). Potential false positive GO terms were filtered. GO, gene ontology.

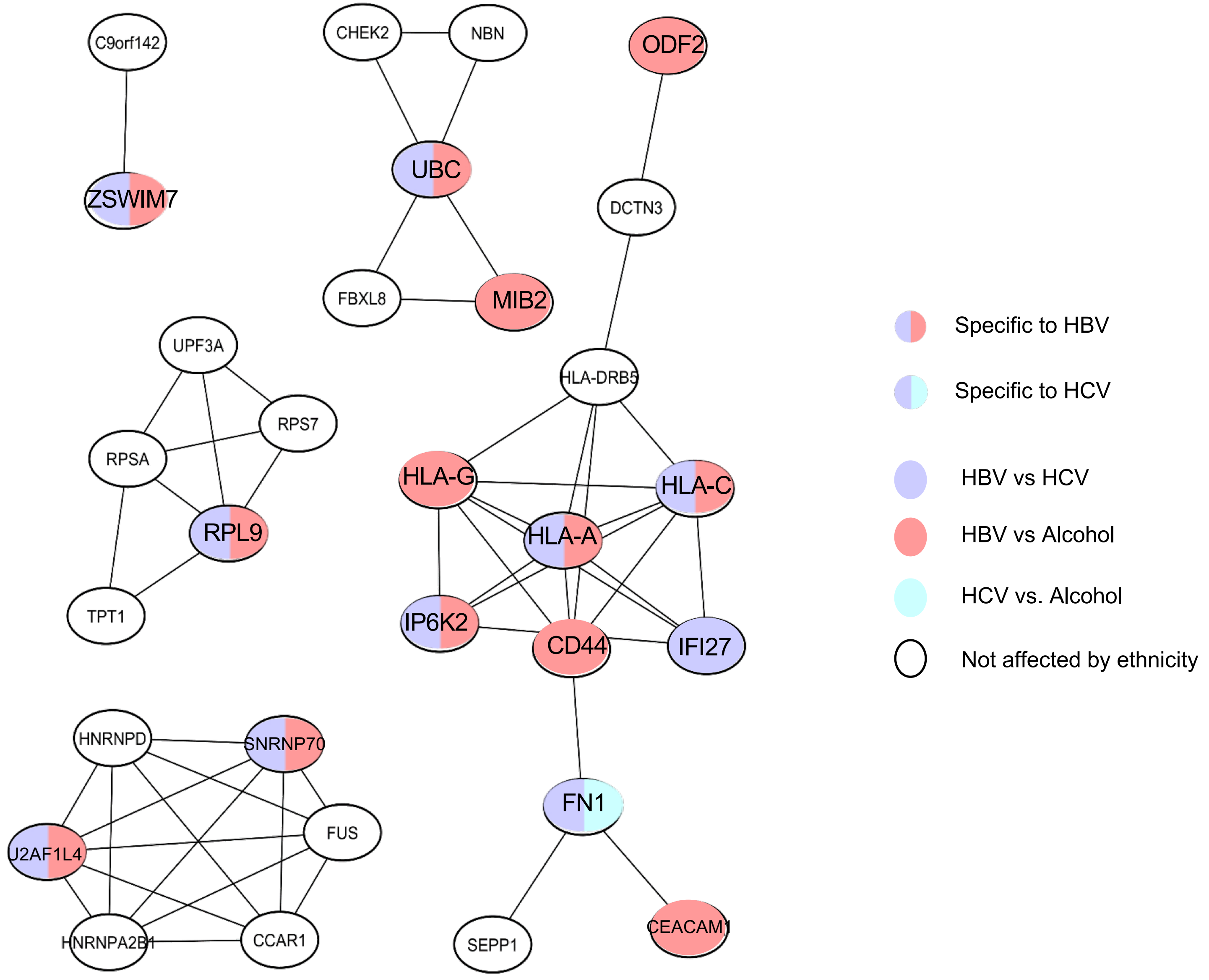

Figure S4. PPI not affected by ethnic differences. Protein-protein interaction (PPI) network analysis was conducted for 198 genes (FDR <0.05, ΔPSI >0.05) differentially associated with AS events in Caucasians and Asians. Of the 30 networked genes from the three etiology group comparisons (Figure 3), 15 genes (colored) were affected by ethnic differences and 15 genes (non-colored) were not affected by ethnicity.

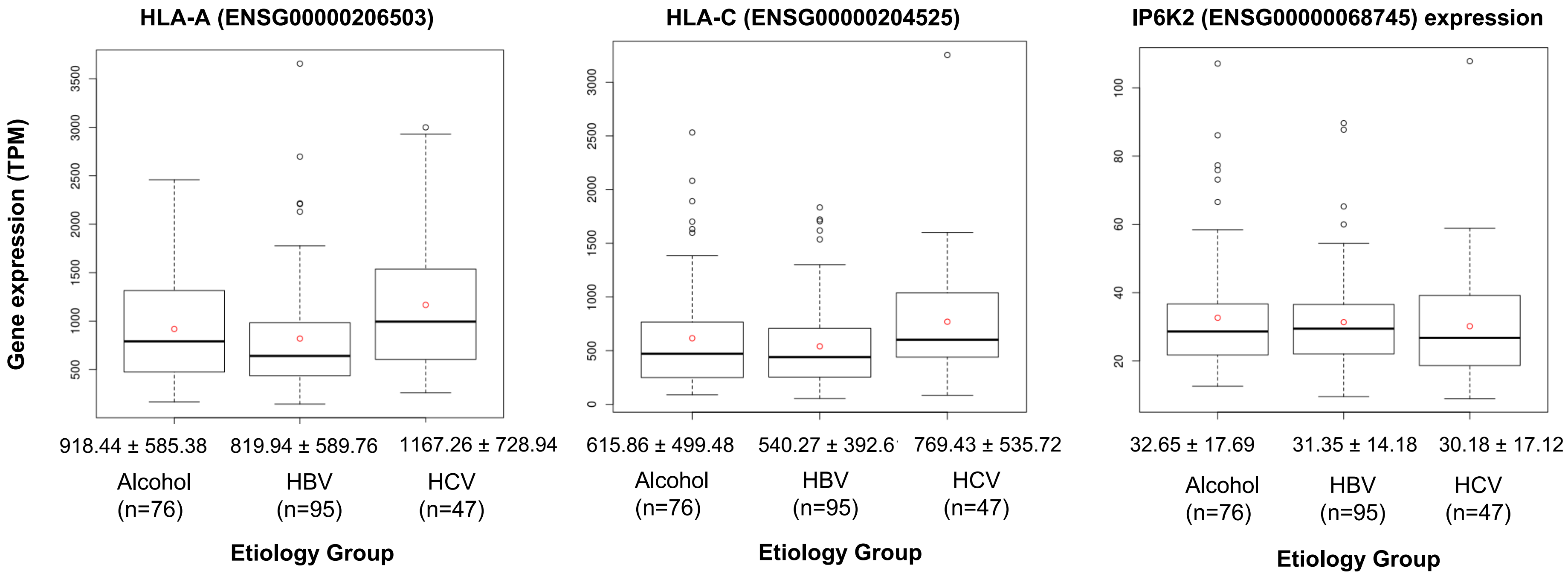

Figure S5. Boxplots for distributions of gene expression among three etiologic groups for our three case studies.

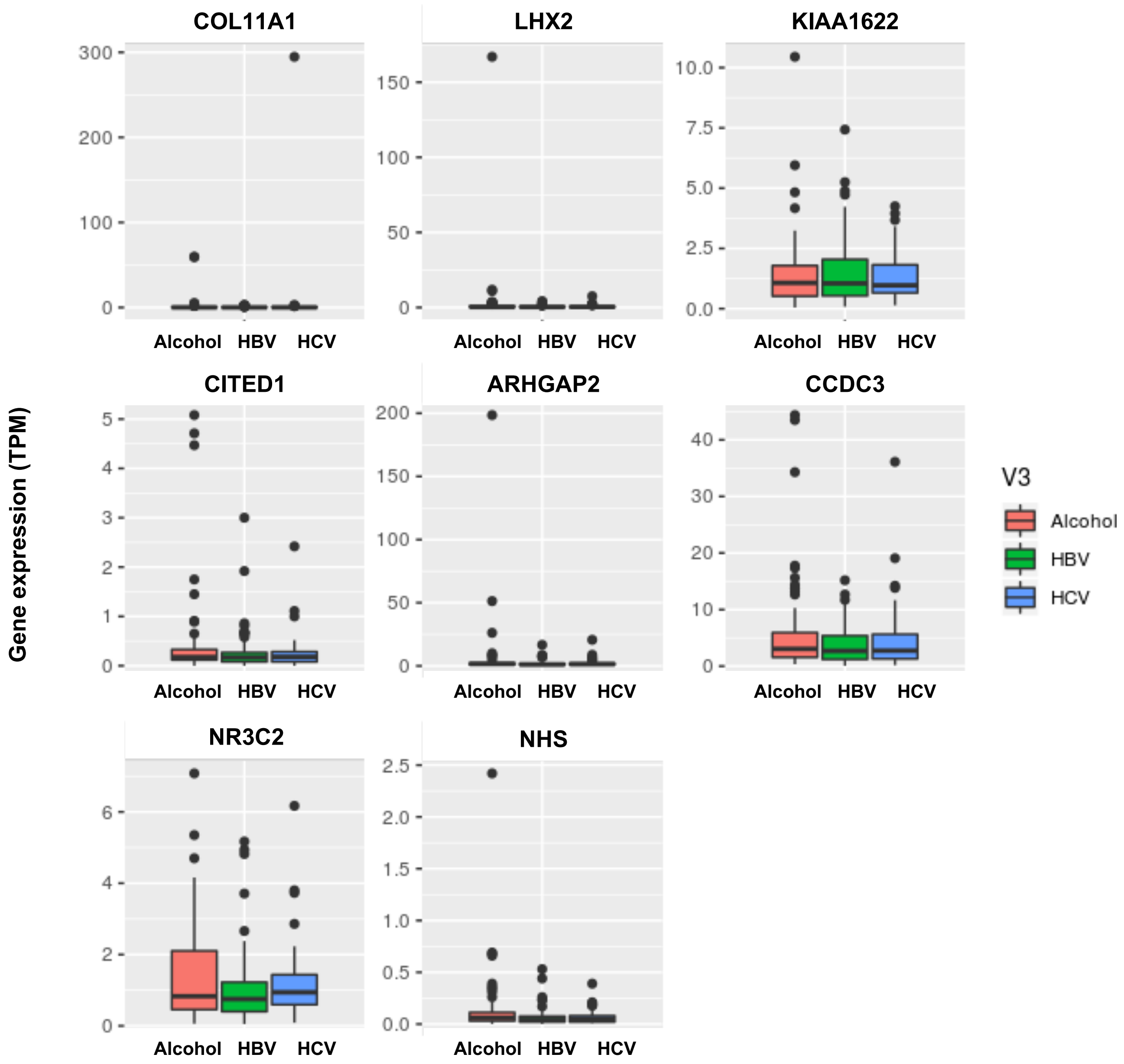

Figure S6. Boxplots for distributions of gene expression among three etiologic groups. All of eight genes are reported to be affected by *UPF3A* via NMD process.

Table S1. Baseline clinical characteristics of study subjects.

Variables (n=218)	Groups			*p*-value*
	**HBV group (n= 95, 43.6%)**	**HCV group (n=47, 21.1%)**	**Alcohol group (n=76, 34.9%)**	
Age (year)§	54±12[a]	61±9[b]	64±11[b]	<0.05
Gender (male), n (%)	78 (82.1)	38 (80.9)	64 (84.2)	0.92
Race, n (%)				<0.05+,
Caucasian	7 (7.4)	30 (63.8)	48 (63.2)	
Asian	84 (88.4)	6 (12.8)	24 (31.6)	
Others	4 (4.2)	11 (23.4)	4 (5.3)	
Albumin (g/dL)[p]	3.8±1.0	3.4±1.1	3.9±0.9	0.08
Total bilirubin(mg/dL)[q]	0.8±0.5	0.9±0.7	0.9±1.2	0.70
PT, INR[r]	1.0±0.1	1.1±0.1	1.1±10.1	0.35
CTP class, A/B, n (%)[s]	87/5 (94.6/5.4)	30/5 (85.7/14.3)	24/2 (92.3/7.7)	0.25
AFP (ng/ml)[m]	$7,4x10^3±2.9x10^4$	$552±2.1x10^3$	$1.1x10^4±3.9$ $x10^4$	0.11
LC, presence, n (%)†	2 (2.1)	10 (21.3)	32 (42.1)	<0.05+,
AJCC (7th)[n]				<0.05+
I	63 (67.7)	26 (60.5)	26 (39.4)	
II	19 (20.4)	11 (25.6)	15 (22.7)	
III (A,B, or C)	9 (9.7)	6 (14.0)	25 (37.9)	
IV (A or B)	2 (2.2)	0 (0)	0 (0)	

Abbreviation: AFP, Alpha-fetoprotein; HBV, hepatitis B virus; HCV, hepatitis C virus; PT, prothrombin time; CTP, Child-Turcotte-Pugh, LC, liver cirrhosis; AJCC,

†, pathologically defined based on Ishak fibrosis score system

Data for p, q, r, s, m, and n were available for 182,182,177, 138, 176, and 202 patients, respectively.

§, mean±standard deviation

*, *P* values were calculated using the ANOVA test (using post hoc Tukey's b test), the *Chi*-square test, or Fisher's exact test, as appropriate.

+, Fisher's exact test

Table S2. Identification of alternative splice events in the three pairwise comparisons of HBV vs. HCV, HCV vs. alcohol, and HBV vs. alcohol.

**Group** / **Count of events**	**Comparison between groups (FDR <0.05, PSI > 0.05)**		
	**HBV *vs.* HCV**	**HBV *vs.* Alcohol**	**HCV *vs.* Alcohol**
Skipped Exon	48	31	16
Retained Intron	14	15	3
Mutually exclusive exon	55	39	7
Alternative 3' site	7	2	2
Alternative 5' site	9	6	1
Total AS events	133	93	29
Genes associated with total AS events	89	69	23

Abbreviations: AS, Alternative Splicing; FDR, false discovery rate; PSI, percent spliced in; HBV,

**Group** / **Count of events**	**Comparison between groups (FDR <0.05, PSI > 0.1)**		
	**HBV *vs.* HCV**	**HBV *vs.* Alcohol**	**HCV *vs.* Alcohol**
Skipped Exon	16	8	2
Retained Intron	4	2	1
Mutually exclusive exon	4	3	1
Alternative 3' site	2	0	1
Alternative 5' site	3	2	0
Total AS events	29	15	5
Genes associated with total AS events	22	11	5

hepatitis B virus; HCV, hepatitis C virus.

Detail result of significantly identified AS exons. Compared between each etiology group (i.e. HBV vs HCV, HCV vs Alcohol, and Alcohol vs HBV). Five different alternative splicing type (ES: Exon skipping, IR: Intron retention, A3SS: alternative 3' splice site, A5SS: Alternative 5' Splice site, and MXE: Mutually exclusive exon) was calculated in each group comparisons. Splicing events with lower than 10% PSI difference between two groups and FDR value was larger than 0.05 was excluded. In addition, to obtain larger gene sets for the further analysis, splicing events lower than 5% PSI difference between two groups and FDR-value was larger than 0.05 was excluded.

Table S3. The demographic summary of Caucasian and Asian population.

Variables (n=199)	Race		*p*-value*
	**Caucasian (n= 85, 43.6%)**	**Asian (n=114, 21.1%)**	
Age (year)§	65±11	56±10	<0.01
Gender (male), n (%),	64 (75.3)	99 (86.8)	0.04
Etiology			<0.01
HBV	7 (8.2)	84 (73.7)	
HCV	30 (35.3)	6 (15.3)	
Alcohol	48 (56.5)	24 (21.1)	
AFP	$1,0x10^4$±$3.7x10^4$	$4.9x10^3$ ±$2.4x10^4$	0.28
LC, presence, n (%)†	17 (20.0)	21 (18.4)	0.86

Abbreviation: AFP, Alpha-fetoprotein; HBV, hepatitis B virus; HCV, hepatitis C virus; LC, liver cirrhosis

†, pathologically defined based on Ishak fibrosis score system

§, mean±standard deviation

*, *P* values were calculated using the student *t*-test or the *Chi*-square test.

Table S4. Identification of alternative splice events in comparison of Caucasian and Asian population.

Count of events	Group comparisons	
	**Caucasian vs. Asian (FDR <0.05, PSI > 0.1)**	**Caucasian vs. Asian (FDR <0.05, PSI > 0.05)**
Skipped Exon	19	73
Retained Intron	5	29
Mutually exclusive exon	7	238
Alternative 3' site	0	13
Alternative 5' site	2	10
Total AS events	33	363
Genes associated with total AS events	25	198

Abbreviations: AS, Alternative Splicing; FDR, false discovery rate; PSI, percent spliced in.

Five different alternative splicing type (ES: Exon skipping, IR: Intron retention, A3SS: alternative 3' splice site, A5SS: Alternative 5' Splice site, and MXE: Mutually exclusive exon) was calculated in race group. To obtain larger gene sets for the further analysis, splicing events lower than 5% PSI difference between two groups was excluded.

Table S5. List of frequently altered genes by etiology			
(FDR < 0.05, ΔPSI>0.05)			
**Common in all etiology**	**Common in HBV**	**Common in HCV**	**Common in alcohol**
CD46	DYRK4	GPALPP1	APLP2
	SMARCC2	FN1	INF2
	PIGQ	IL17RC	C8orf44
	PDXDC2P	CYB5A	
	ALDH3A2	HAAO	
	CIRBP	RP4-665J23.1	
	ADAM15		
	S100A13		
	IP6K2		
	C8orf59		
	UBC		
	ZSWIM7		
	U2AF1L4		
	HLA-A		
	HLA-C		
	SLC25A37		
	CPNE1		
	SNRNP70		
	NDUFS5		
	ULK3		
	IL32		
	MST1P2		
	MST1L		
	TRNAU1AP		
	HNRNPD		
	RPL9		
	GTF2I		
(FDR < 0.05, ΔPSI>0.1)			
**Common in all etiology**	**Common in HBV**	**Common in HCV**	**Common in alcohol**
NA	PIGQ	CYB5A	NA
	ALDH3A2	RP4-665J23.1	
	ADAM15		
	IP6K2		
	HLA-C		
	SLC25A37		
	C8orf59		
